# Assessment of the current state of knowledge and risk factors of cervical cancer among women in the Buea Health District, Cameroon

**DOI:** 10.11604/pamj.2019.33.38.16767

**Published:** 2019-05-21

**Authors:** Ngwayu Claude Nkfusai, Samuel Nambile Cumber, Judith K Anchang-Kimbi, Kah Emmanuel Nji, Joyce Shirinde, Nota Damian Anong

**Affiliations:** 1 Department of Microbiology and Parasitology, Faculty of Science, University of Buea, Buea, Cameroon; 2 Cameroon Baptist Convention Health Services (CBCHS), Yaoundé, Cameroon; 3 Institute of Medicine, Department of Public Health and Community Medicine (EPSO), University of Gothenburg, Box 414, SE - 405 30 Gothenburg, Sweden; 4 Faculty of Health Sciences, University of the Free State, Bloemfontein, South Africa; 5 School of Health Systems and Public Health, Faculty of Health Sciences, University of Pretoria Private Bag X323, Gezina, Pretoria, 0001, Pretoria, South Africa; 6 Department of Zoology and Animal Physiology, Faculty of Science, University of Buea, Buea, Cameroon; 7 Department of Public Health and Hygiene, Faculty of Health Sciences, University of Buea, Buea, Cameroon; 8 Department of Biological Sciences, Faculty of Science, University of Bamenda, Bamenda, Cameroon

**Keywords:** Cervical cancer, women, knowledge, risk factors, Buea Health District

## Abstract

**Introduction:**

Cervical cancer is a malignant proliferation of the cells of the uterine cervix and can be treated if diagnosed earlier. It is the second most common gynecological malignancy worldwide and the leading cause of cancer associated mortality among women in Africa and Cameroon. This study sort to determine the current state of knowledge of cervical cancer and its risk factors in the Buea Health District of the South West Region of Cameroon.

**Methods:**

This was a cross-sectional community based survey. We recruited 433 eligible women, in four (4) Health Areas (Molyko, Bolifamba, Muea and Buea Town) of the Buea Health District and used validated and pre-tested questionnaires to collect data. Collected data were keyed into Epi info version 7.2 statistical software and exported to SPSS Version 25 for analysis. Level of significance was set at P-value < 0.05.

**Results:**

Fifty eight percent (58%) of the participants had good knowledge of cervical cancer. 58.99% (95%CI = 54.30-63.52) had good knowledge on the risk factors of cervical cancer. 40% knew at least one of the following risk factors; cigarette smoking, many sexual partners, family history of cervical cancer, being HIV/AIDS positive and giving birth 5 or more times. There was a significant association, OR = 7.5; 95%CI = 2.14-26.33; P = 0.001; X^2^ = 11.4 between having heard of cervical cancer and having “good” knowledge of cervical cancer among women in Buea.

**Conclusion:**

Most of the women had heard of cervical cancer but the knowledge of the risk factors of cervical cancer among women aged 18-68 years in the Buea Health District is low. We found no association between awareness and knowledge of risk factors among the women.

## Introduction

Cervical cancer is cancer of the lower portion of the uterus, just above the vagina and it is a malignant proliferation of the cells of the uterine cervix [1]. Cervical cancer is a preventable and curable disease which can be treated if diagnosed early. The causative agent of cervical cancer is the Human Papilloma Virus (HPV). HPV is transmitted through sexual intercourse and can later cause cervical cancer through a slow growth over a period of 10-20 years [1]. Highly Active Antiretroviral Therapy (HAART) and evolution of cervical disease causes a high prevalence of cervical cancer in Cameroon [2]. It has been shown that, at least 50% of women who are sexually active have suffered from an infection with at least one strain of HPV [3, 4]. However, most of these cases do not progress to the disease state because the immune system fights against its progression. The global incidence of cervical cancer is greater than 530 000 annually, with death approaching 275 000 per year [5]. The prevalence of cervical cancer worldwide is estimated by [6] to be 12%. Cervical cancer is the second most commonly diagnosed cancer after breast cancer and the third leading cause of cancer death among females in less developed countries. Incidence rates are the highest in countries with poverty. About 90% of cervical cancer deaths occurred in developing parts of the world [7]. One of the most important reasons for the incidence of cervical cancer in developing countries is the lack of early detection of pre -cancerous lesions and treatment of the lesions before they progress to the disease state. Among the newly diagnosed cases, 86% are reported in poor countries; also, 88% of deaths resulting from cervical cancer is in the low-income countries [8]. In Africa, the incidence is 80 000 per annum, with an annual mortality rate of 75%; with most cases in sub-Saharan Africa [8]. In Cameroon, cervical cancer prevalence was shown to be up to 13.8%; based on a study carried out in the capital city Yaoundé by [9]. More than 6 million Cameroonian females who are aged 15 and above are at risk of developing cervical cancer. There are 1993 new cases of cervical cancer yearly of which 1120 die of the disease [3]. In poor countries, awareness as well as uptake of cervical cancer screening services has remained poor over the years. Several studies done in communities and among women in sub-Saharan Africa revealed that knowledge was generally poor [10]. Risk factors of cervical cancers have also been highly demonstrated among Cameroonian women, especially the rural women [11]. Mogtomo and colleagues [12] have demonstrated a high incidence of sexually transmissible infections, multiple sexual partners, low use of condoms and other risk factors of cervical cancer among students in the University of Douala [13]. This therefore, calls for relevant measures to reduce this trend of progression. With current and appropriate measures to prevent cervical cancer, success of screening will largely depend on the awareness and beliefs among women [13]. The level of knowledge of the general population is very important in determining the right strategy in planning an effective intervention against cervical cancer. WHO reported that cervical cancer screening coupled with immediate management leads to early detection of precancerous and cancerous cervical lesions, thus preventing serious morbidity and mortality due to the disease [2]. In a study carried out by [14], they showed that the level of awareness of HPV infection and prevention of cervical cancer is moderately low in Cameroon. The level of awareness of cervical cancer and its risk factors is low in Cameroon as seen in studies done by [14] in 2013. No studies have been done in a community level in the Buea Health District on the assessment of knowledge of cervical cancer and its risk factors, this study therefore, aimed to determine the current state of knowledge of cervical cancer and its risk factors in the Buea Health District, South West Region of Cameroon.

## Methods

### Study design, sitting and population

This study was a cross-sectional community based survey in the Buea Health District of the South West Region of Cameroon. This study was conducted in the Buea Health District, Fako Division of the South West Region of the Republic of Cameroon. Buea is the capital of the South West Region with coordinate’s 4°N 9°E of the Greenwich meridian. It is located on the eastern slopes of mount Cameroon and has a population of about 200,000 inhabitants.

### Selection criteria

**Inclusion criteria:** women aged between 18 years and above who have been residing in the Buea Health District for at least two years; those who meet the above criteria and were willing to take part in the study voluntarily.

**Exclusion criteria:** women who did not give consent to participate; women who wanted to be motivated before they responded to the questionnaire; women who were younger than 18 years of age.

### Sample size and sampling

Sample size of women on current state of knowledge as well as risk factors in the South West Regions was determined using the Fisher’s formula:

$$N=\frac{z^{\text{2}}*P(P-1)}{e^{2}}$$

N = minimum sample size, Z = Z - value corresponding to 95% confidence interval (1.96), P = The level of knowledge of cervical cancer and its risk factors in the South West Regions of Cameroon is not known; hence 50 % will be used. e = error margin (0.05).

$$N=\frac{1.96^{2}*0.05(1-0.05)}{0.05^{2}}$$

Therefore,

N = 384.

From the calculation, a sample size of 384 participants were to be administered in the Buea Health District, 96 questionnaires administered in each of the four selected health areas (Molyko, Buea Town, Bolifamba and Muea). The questionnaires were administered at home as it was a door to door study.

### Administration questionnaires

Questionnaires were administered among consented participants in their homes who agreed to participate in the study. The questionnaires captured data on socio-demographic characteristics, awareness level of cervical cancer and knowledge on its risk factors.

### Data collection

This was a community based survey where all participants who consented were interviewed using a structured questionnaire filled in their homes. Prior to use in the study participants, a total of 20 questionnaires were pretested at the University of Buea among female students aged 18 years and above with the aim of revising poorly structured questions, estimate the average time required to fill the questionnaire and thus validate the use of the questionnaire in our context. It was estimated that; each questionnaire could be administered for 30-45 minutes after the pretest. A total of 433 questionnaires were administered to women greater than or equal to 18 years of age resident in the Buea Health District for a period of 2 months (July-August) to assess their level of awareness of cervical cancer and their knowledge level on its risk factors. Knowledge on cervical cancer consisted of 12 questions while knowledge on risk factors of cervical cancer consisted of 8 questions which were: 1) Does consistent use of condom or diaphragm help prevent HPV infection and cervical cancer? 2) Cigarette smoking is a risk factor for cervical cancer? 3) Early first sexual intercourse is a risk factor for cervical cancer? 4) Having many sexual partners put you at risk of developing cervical cancer in future? 5) Family history of cervical cancer is a risk factor for cervical cancer? 6) Sexually transmitted infections are risk factors for cervical cancer? 7) Giving birth to five or more children increases ones’ risk of developing cervical cancer? 8) Being HIV positive increases the chances of developing cervical cancer?

Each correct response was scored as 1 and 0 for a wrong response The knowledge scores for an individual was calculated and summed up to give a total knowledge score on 12 and the knowledge score for risk factors of cervical cancer for each individual was calculated and summed up to give a total score of 8. A score between 0-4 was classified as poor, 5-8 as good and 9-12 as excellent for knowledge of cervical cancer, while a score between 0-3 was classified as poor, 4-6 as good and 6-8 as excellent for knowledge on risk factors of cervical cancer. This was adapted from a study conducted by [15].

### Data analyses

Data were entered into a template designed in Epi info. The data were verified for completion and incomplete entries were deleted, cleaned and exported to SPSS version 25 for analyses. Descriptive analysis was carried out by calculating the mean and frequencies of different variables Chi-Square test was used to determine associations between categorical variable. Statistical significant was set at P-value < 0.05 Results were presented in the form of frequency tables and charts.

### Ethical and administrative consideration

Ethical clearance was obtained from the Institutional Review Board of the Faculty of Health Sciences, University of Buea. Administrative clearance was obtained from the Regional Delegation of Public Health for South West Region Cameroon. Participants had the study protocol carefully explained to them and participation was voluntary. Written informed consent was obtained from all participants.

## Results

### Demographic characteristics

We approached over 500 women in 4 Health Areas of the Buea Health District. However, a total of 433 participants aged 18 and above who met the study criteria, and gave their consent were recruited within a 4-week study period. The average age of the participants was 30.5 years. Most of the participants were married (60.7%), Farmers (46.7%) and Christians (98.8%). Half (50.8%) of the participants were aged between 35 to 51 years (50.8%) and only 24% of them had completed the high school (Table 1).

**Table 1:** Socio-demographic characteristics of the participants, N = 433

Characteristics		Frequency (F)	Percentage (%)
**Age Category**			
	18-34	141	32.6
	35-51	220	50.8
	52-68	72	16.6
**Level of education**			
	No formal	6	1.4
	Primary	174	40.2
	Secondary	149	34.4
	Tertiary	104	24
**Marital status**			
	Married/Separated/Widow	170	39.3
	Single	263	60.7
**Religion**			
	Christian	428	98.8
	Muslim	5	1.2
**Occupation**			
	Business	129	29.8
	Farmers	202	46.7
	Others	102	23.5

N= Sample size

### Knowledge of participants on cervical cancer

Table 2 gives a detailed representation of the participants’ responses to knowledge related questions on cervical cancer. About the findings indicate that 50.7% (95% CI = 46% - 55.37) of the study population had good knowledge on cervical cancer and 49.3% had poor knowledge of the disease.

**Table 2:** Knowledge of the participants on cervical cancer

	Yes F(%)	No F(%)	Don’t know F(%)
Knowledge on cervical cancer			
Have you heard of cervical cancer	251(57.8)	183(42.2)	
First heard of cervical cancer from social media	295(68.02)	138(31.98)	
Is cervical cancer the 1^st^ cause of death in women in Cameroon	183(42.2)	41(9.4)	210(48.4)
Is cervical cancer caused by a virus	131(30.2)	28(6.5)	275(63.4)
Have you heard about HPV	100(23.0)	334(76.9)	
Do patients always show symptoms	50(11.5)	76(17.5)	308(71.0)
Can cervical cancer be prevented via vaccination	234(53.9)	22(5.1)	178(41.0)
Is cervical cancer curable	192(44.2)	46(10.6)	196(45.2)

**F =** Frequency**, % =** Percentage

### Mortality associated with cervical cancer in Cameroon

Only 42.2% (183 participants) knew that cervical cancer was the most common cause of cancer deaths among all gynaecological cancers in Cameroon.

### Causative agent of cervical cancer

About one-third (30.2%) of the total participants rightly said that the causative agent is a virus.

**Determining the knowledge of the participants on the risk factors of cervical cancer**

### Knowledge of risk factors of cervical cancer

In Table 3, the frequencies beside each risk factor represent the number of participants who rightly considered the said variable as a risk factor of cervical cancer.

**Table 3:** Knowledge of the participants on the risk factors of cervical cancer

	Yes F(%)	No F(%)	Don’t know F(%)
Risk factors of cervical cancer			
Does consistent use of condom or diaphragm prevent cervical cancer	137(31.6)	42(9.7)	255(58.8)
Is cigarette smoking a risk factor	171(39.4)	30(6.9)	233(53.7)
Is early 1^st^ sexual intercourse a risk factor	138(31.8)	36(8.3)	260(59.9)
Is having several sexual partners a risk factor	236(54.4)	7(1.6)	191(44.0)
Is having a family history of cervical cancer a risk factor	55(12.7)	132(30.4)	247(56.9)
Having an STD is a risk factor	213(49.1)	10(2.3)	211(48.7)
Being HIV/AIDS positive is a risk factor	244(56.2)	94(2.1)	181(41.7)
Is giving birth 5 or more times a risk factor	56(12.9)	86(19.9)	292(67.3)

**F** = Frequency, **%** = Percentage

Using descriptive statistics, about 58.99% (95%CI=54.30-63.52) had good knowledge on the risk factors of cervical cancer

### Participant’s knowledge knowledge on the risk factors of cervical cancer

The study revealed that 58.99% (95% CI = 54.30 - 63.52) of participants had good knowledge on the risk factors of cervical cancer while 41/01% had poor knowledge.

**Determining the percentage of the population who have screened for cervical cancer and the prevalence of cervical cancer in the screened population**

### Percentage of the population who had screened for cervical cancer

Only 15.51% (95% CI = 80.77% - 87.60%) of the study participants had screened for cervical cancer while up to 84.49% had never screened for cervical cancer.

### Prevalence of cervical cancer in the screened population

The prevalence of cervical cancer in the screened population was 28.4% (95% CI = 18.01% - 40.69%) and 71.64% from the screened population were negative.

### Association between family history and cervical cancer

A chi-square test showed that there was a statistically significant association (OR = 7.5; 95% CI = 2.14-26.33; P = 0.001; X^2^ = 11.4 between family history of cervical cancer and the prevalence of cervical cancer (Table 4).

**Table 4:** Comparing family history of cervical cancer with the prevalence of cervical cancer

		Cervical cancer	
**Family history**		**Non-reactive (%)**	**Reactive (%)**
	**No (%)**	32(88.89)	4(11.11)
	**Yes (%)**	16(51.61)	15(48.39)

X^2^(Chi-square value) =11.4, P-value =0.001

## Discussion

This cross-sectional community based study was conducted to assess the knowledge of cervical cancer and risk factors among women aged 18-68 years in the Buea Health District and to evaluate if there is any difference in the level of knowledge of cervical cancer and risk factors of cervical cancer among women in the Buea Health District. In our study, we recruited 433 participants. Our age range was 18 to 68 years, which was similar to [15], in Nigeria, with an age range of 16 to 65 years. The mean age of the study population was 30.5 years, with a modal age of 21 years. However, this was higher than the mean age of 21.5 years obtained by [16-18] in South Africa. This difference could be due to the fact that [16], studied only younger population of females. Majority of our participants (98.8%) were Christians and single; this was similar to other studies carried out in other parts of Africa. Findings indicate that 57.8% of the participants had heard of cervical cancer in the past. This is slightly lower than that carried out by [17] in schools and clinics in the North West Region of Cameroon which revealed an awareness of cervical cancer, of above 70%. This could be due to the large sample size in their study. Furthermore, [17] carried out an exploratory study in the North West Region of Cameroon on awareness, knowledge and beliefs on cervical cancer among nurses and found out that 39.1% had a low knowledge of cervical cancer. This is slightly higher than our findings where 42.2% of our participants had a low knowledge of cervical cancer.

The high awareness of cervical cancer among these women could be due to the fact that they were within an urban environment, and thus can easily access information from the internet, mass media, and press prints. Our findings were however higher than the 33% obtained by [17] among female undergraduate students in South Africa. This difference could be due to the fact that [18], recruited only first year undergraduate students who could be considered to have less knowledge on cervical cancer compared to their senior counterparts. Most our study participants had heard about cervical cancer from mass media. This was similar to results obtained by [19] in Lagos Nigeria and by [20] in Ghana. In spite of the high awareness of cervical cancer among citizens of Buea, their overall knowledge on cervical cancer and its risk factors was low [10]. In Kampala Uganda revealed a lower knowledge of cervical cancer among participants. Our findings were also similar to [21] in Buea Cameroon, where up to 42.2% of participants had poor knowledge on cervical cancer. Low knowledge was also reported by [14] in the Far North region of Cameroon among women. These similarities could be due to the fact that these studies were done in low income countries with similar characteristics and behaviours. With regards to HPV, only 23% of participants had heard about Human Papilloma Virus (HPV). This was low compared to the 54% obtained in Portugal. This discrepancy in findings could be attributed firstly to the fact that Portugal is a high-income country that has implemented multiple cervical cancer health programs. Secondly, more than half of their participants were Health Science students. Our respondents had a low knowledge about a link between HPV and cervical cancer, as up to three quarter of them didn’t know that HPV was the cause of cervical cancer. This was in line with reports from other parts of Africa. Low knowledge on the risk factors of cervical cancer was also noticed among our participants. More than half of the participants didn’t know most of the risk factors of cervical cancer. We had much similarities with the findings of Medeiros, [22] where 62% identified multiple sexual partners as a risk factor of cervical cancer, early sexual intercourse and smoking, our findings were however higher than that obtained by [16] in South Africa where only 22% identified multiple sexual partner as a risk factor, early sexual intercourse by 19%, and smoking by 10%. This difference could be due to the fact that [16] surveyed more naïve first year university students. Less than 20% of our participants identified multiple deliveries as a risk factor of cervical cancer, and family history was identified by 12.7% of participants. This mirrored the findings of [21] in Buea were family history and multiple deliveries were considered a risk of cervical cancer by less than 50% of participants. This similarity could be due to their similar study area. Worse case scenarios were reported among women in a community in Ghana [23], where 93.6% of participants had no knowledge on the risk factors of cervical cancer. This situation could possibly be due to the fact that a significant number of their participants were petty traders and fishmongers who had not attained beyond secondary level of education. The prevalence of cervical cancer in the screened population was 28.4%. This is higher than the 13.8%; based on a study carried out by [9] Yaounde, capital city of Cameroon.

**Some limitations in our study include:** the study did not recruit participants from all the Health Areas of the Buea Health District. Furthermore, studying self-reported knowledge is itself a limitation because one cannot rely totally on the information provided by the participants because of recall bias and social desirability bias. Despite these shortcomings, this study provides relevant information in the context of very limited epidemiological data on cervical cancer in the Buea Health District of the South West Region of Cameroon, especially among women in the semi-urban milieu.

## Conclusion

This study investigated the knowledge level of cervical cancer and its risk factors of Cervical cancer in the Buea Health District. From the results obtained, it can be concluded that most (57.8%) of the participants had good knowledge and a high level of awareness of cervical cancer in the Buea Health District. Also, more than 60% of the participants did not know most of the risk factors (cigarette smoking, many sexual partners, family history of cervical cancer, being HIV/AIDS positive and giving birth 5 or more times) of cervical cancer. This study found a significant association between family history of cervical cancer and the prevalence of cervical cancer. Furthermore, the prevalence of cervical cancer in the screened population was low, 28.4% and there was no association between awareness and risk factors among the women.

### What is known about this topic

- A cross-sectional study was conducted in 6 regions in Cameroon to determine the prevalence of cervical premalignant lesions;
- Assessment of awareness and preventive behaviours of cervical cancer among female university students in South Africa; infection and multiple sexual partners are risk factors of cervical cancer;
- A study was carried out in schools and clinics in the North West Region of Cameroon revealed an awareness of cervical cancer, preventive measures, screening method and HPV vaccination and a study among health workers in Cameroon and 75% of the respondents agreed that the general public is not sufficiently informed about cervical cancer.

### What this study adds

- The study revealed a good knowledge level (58%) of cervical cancer awareness among women in the Buea Health District though lower than the national level thus indicating that awareness should be scaled up in rural areas so as to reduce the morbidity and mortality as a results of the disease;
- The study shows that the level of awareness of risk factors was low so there is need therefore to put more emphasis on educating and creating awareness among communities about cervical cancer, risk factors, signs and symptoms in all the health areas of the Buea Health District;
- The prevalence of cervical cancer in the screened population was up to 28.4%: this is shows there is need for sensitization on the disease and its risk factors so as to reduce the prevalence; furthermore, there was an association between family history and the prevalence of the disease; so, sensitization on the disease and its risk factors remains paramount in reducing the prevalence of the disease.

## Competing interests

The authors declare no competing interests.

## References

[1] Daniel A, Aruna P, Ganesan S, Joseph L (2017). Biochemical assessment of human uterine cervix by micro-Raman mapping. Photodiagnosis Photodyn Ther.

[2] World Health Organization (2013). WHO guidelines for screening and treatment of precancerous lesions for cervical cancer prevention: supplemental material: GRADE evidence-to-recommendation tables and evidence profiles for each recommendation.

[3] Coste J, Cochand-Priollet B, de Cremoux P, Le Galès C, Isabelle C, Vincent M, Labbé S, Vacher-Lavenu MC, Vielh P (2003). Cross sectional study of conventional cervical smear, monolayer cytology, and human papillomavirus DNA testing for cervical cancer screening. BMJ.

[4] DeGregorio GA, Bradford LS, Manga S, Tih PM, Wamai R, Ogembo R, Sando Z, Liu Y, Schwaiger C, Rao SR, Kalmakis K (2016). Prevalence, predictors, and same day treatment of positive VIA enhanced by digital cervicography and histopathology results in a cervical cancer prevention program in Cameroon. PLoS One.

[5] Kafuruki L, Rambau PF, Massinde A, Masalu N (2013). Prevalence and predictors of cervical intraepithelial neoplasia among HIV infected women at Bugando Medical Centre, Mwanza-Tanzania. Infect Agent Cancer.

[6] Saslow D, Solomon D, Lawson HW, Killackey M, Kulasingam SL, Cain J, Garcia FA, Moriarty AT, Waxman AG, Wilbur DC, Wentzensen N (2012). American Cancer Society, American Society for Colposcopy and Cervical Pathology, and American Society for Clinical Pathology screening guidelines for the prevention and early detection of cervical cancer. CA: Cancer J for clinicians.

[7] Torre LA, Bray F, Siegel RL, Ferlay J, Lortet-Tieulent J, Jemal A (2015). Global cancer statistics, 2012. CA Cancer J Clin.

[8] Lim JN, Ojo AA (2017). Barriers to utilisation of cervical cancer screening in Sub Sahara Africa: a systematic review. Eur J Cancer Care (Engl).

[9] Enow Orock GE, Ndom P, Doh AS (2012). Current cancer incidence and trends in Yaounde, Cameroon. Oncol Gastroenterol Hepatol Reports.

[10] Nakibuule C (2014). Knowledge, attitudes and practices regarding cervical cancer among women and men in Kampala City, Uganda.

[11] Tufon EN, Yuwun Novert B, Egba S, Ndohnui NN (2011). Prevalence, associated risk factors and methods of diagnosing cervical cancer in two hospitals in Yaounde, Cameroon.

[12] Mogtomo ML, Malieugoue LC, Djiepgang C, Wankam M, Moune A, Ngane AN (2009). Incidence of cervical disease associated to HPV in human immunodeficiency infected women under highly active antiretroviral therapy. Infect Agent Cancer.

[13] Koanga MM, Ngono NA, Wandjis A, Dongang NR, Donfack TH, Nganwa G, Wankam M, Amvam ZP (2010). Sexual behaviour: human papilloma virus and cervical cancer risk among university students in cameroon. Afri J Haematol & Oncol.

[14] Abongwa LE, Clara AM, Edouard NA, Ngum NH (2015). Sero-Prevalence of human immunodeficiency virus (HIV) and Hepatitis B virus (HBV) Co-Infection among pregnant women residing in Bamenda Health District, Cameroon. Int J Curr Microbiol App Sci.

[15] Owoeye IO, Ibrahim IA (2013). Knowledge and attitude towards cervical cancer screening among female students and staff in a tertiary institution in the Niger Delta. Inter J Med & Biomed Research.

[16] Hoque ME, Ghuman S, Van Hal G (2013). Human Papillomavirus vaccination acceptability among female university students in South Africa. Asian Pac J Cancer Prev.

[17] Wamai RG, Ayissi CA, Oduwo GO, Perlman S, Welty E, Welty T, Manga S, Onyango MA, Ogembo JG (2013). Awareness, knowledge and beliefs about HPV, cervical cancer and HPV vaccines among nurses in Cameroon: an exploratory study. Int J Nurs Stud.

[18] Hoque E, Hoque M (2009). Knowledge of and attitude towards cervical cancer among female university students in South Africa. SAJEI.

[19] Ojesina AI, Lichtenstein L, Freeman SS, Pedamallu CS, Imaz-Rosshandler I, Pugh TJ, Cherniack AD, Ambrogio L, Cibulskis K, Bertelsen B, Romero-Cordoba S (2014). Landscape of genomic alterations in cervical carcinomas. Nature.

[20] Abotchie PN, Shokar NK (2009). Cervical cancer screening among college students in Ghana: knowledge and health beliefs. Int J Gynecol Cancer.

[21] Halle C, Andersen E, Lando M, Aarnes EK, Hasvold G, Holden M, Syljuåsen RG, Sundfør K, Kristensen GB, Holm R, Malinen E (2012). Hypoxia-induced gene expression in chemoradioresistant cervical cancer revealed by dynamic contrast-enhanced MRI. Cancer Res.

[22] Silva J, Cerqueira F, Medeiros R (2014). Chlamydia trachomatis infection: implications for HPV status and cervical cancer. Arch Gynecol Obstet.

[23] Ebu NI, Mupepi SC, Siakwa MP, Sampselle CM (2014). Knowledge, practice, and barriers toward cervical cancer screening in Elmina, Southern Ghana. Int J Womens Health.

